# Gangrenous meckel's diverticulum in a strangulated umbilical hernia in a 42 year-old woman: a case report

**DOI:** 10.1186/1757-1626-3-10

**Published:** 2010-01-07

**Authors:** Ilker Sengul, Demet Sengul, Serhat Avcu, Omer Parlak

**Affiliations:** 1 Department of General Surgery, Giresun University Faculty of Medicine, 28100 Giresun, Turkey; 2 Department of Pathology, Prof Dr A Ilhan Ozdemir State Hospital, 28100 Giresun, Turkey; 3 Department of Radiology, Yuzuncu Yil University Faculty of Medicine, 65100 Van, Turkey; 4 Department of General Surgery, Ankara Ataturk Education and Research Hospital, Ankara, Turkey

## Abstract

**Introduction:**

Meckel's diverticulum affects 1 - 3% of general population and is known as the most common anomaly of gastrointestinal tract. However, its estimated lifetime complication rate is approximately 4%. Intestinal obstruction is most common complication of Meckel's diverticulum in adult population.

**Case presentation:**

In the present study, we reported a 42-year-old female patient with a gangrenous Meckel's diverticulum in a strangulated umbilical hernia sac treated by dissection of diverticulomesenteric bands and diverticulectomy. In 36 months follow-up, there was neither any complication nor recurrence of hernia.

**Conclusion:**

This case represents a gangrenous Meckel's diverticulum in a strangulated umbilical hernia sac diagnosed in case of emergency. Although it is a very rare phenomenon, we should be vigilant for this entity especially in case of emergency.

## Introduction

Firstly in 1700, Littre reported two patients with traction diverticula in an inguinal hernia sac that is known as Meckel's diverticulum today. Also in 1777, a similar case with a hernia including some amount of intestinal wall was falsely reported as same entity. Meckel described the small bowel diverticula now called with his own name in 1800 [1].

Meckel's diverticulum is reported to occur in 1-3% of general population and autopsy series, and it is also known as the most common anomaly of gastrointestinal tract [2,3]. It is found in men more frequently (63%) than women [4,5] and 30% of patients have other anomalies [6]. Although its anatomic localization is not constant, it most often occurs in 100 cm ileal portion, proximal to ileocecal valve [7]. Also, length of this diverticulum changes from 0.5 cm to 85 cm, but mean length is reported as 5 cm [8].

## Case presentation

A 42-year-old female was admitted to our department in December 2006 with complaints of pain and a mass on the umbilical region of her body. She had had a mass increasing in size during past 5 years on the umbilical area and a localized sharp manner pain that its intensity increased especially during past 2 weeks also.

On physical examination; distended abdomen and protruding and deformed umbilicus were detected. The skin of umbilical region was indurated, erythematoused and sensitive. Temperature of the skin on the mass was also increased. A soft, mobile mass of nearly 4 × 3 cm in size on the umbilical region and a longitudinal fascial defect of approximately 3 cm in length including both the umbilicus and the adjacent part of linea alba were palpated. During the operation performed under exigent conditions, umbilical defect was exposed. The part of small intestine that was discoloured to grayish-black of 7 cm in size was determined as Meckel's diverticulum. The fibrous bands of diverticulum associated with intestinal mesentery were dissected and then diverticulectomy and end to end ileoileal anastomosis were performed. Lastly, herniorrhaphy was performed for the fascial defect.

**Figure 1:**
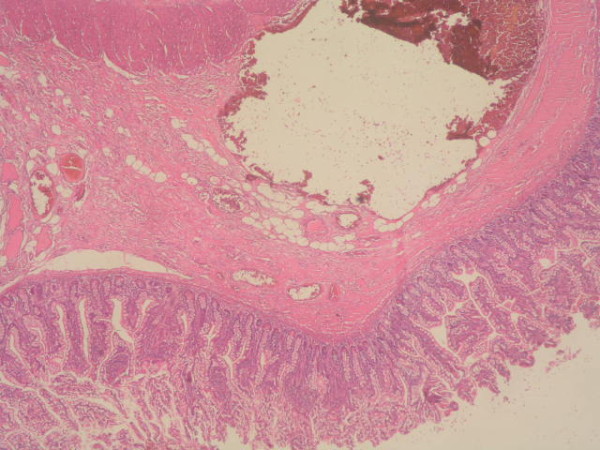
**Congestive vascular structures and massive hemorrhagic findings under the surface epithelium of small bowel (Haematoxylin & Eosin, Original magnification ×40)**.

**Figure 2:**
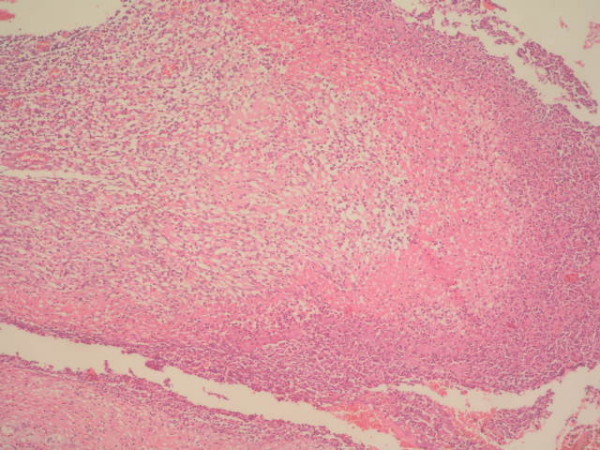
**Massive ulceration on the surface epithelium of small bowel (Haematoxylin & Eosin, Original magnification ×40)**.

Microscopic examinations revealed mucosal infarctions in some places of diverticulum, and diverticular structure was laid down with heterotopic small intestinal mucosa (Figure 1, and Figure 2). Neither complication nor recurrence of hernia was detected during 36 months follow-up.

## Discussion

Incomplete obliteration of omphalomesenteric (vitelline) duct which must be obliterated during 8^th ^week of gestation under normal conditions, results mostly in Meckel's diverticulum. These are true diverticula, because they include all layers of small bowel wall. Microscopically nearly 60% of Meckel's diverticula contain heterotopic mucosa [7]. Perforation, vesicodiverticular fistula, bleeding, ulceration, intestinal obstruction, intussuseption, and tumors are denoted in complications of Meckel's diverticulum [9]. Most common tumor arising from Meckel's diverticulum is carcinoid tumors and its reported incidence is only 2% [10].

Although the lifetime complication rate of the disease is estimated nearly 4%, more than half of patients in whom arising symptoms of complications of that are younger than 10 years old. In adults, most common complication of Meckel's diverticulum is intestinal obstruction; but in children, it is bleeding [7]. In our case, we detected a strangulated, gangrenous Meckel's diverticulum of 7 cm in size in a strangulated umbilical hernia sac and we performed dissection of diverticulomesenteric band, diverticulectomy and end to end ileoileal anastomosis. Histopathological examinations revealed diverticular structure lying down with heterotopic small intestinal mucosa with mucosal infarctions.

Although diverticulectomy and dissection of diverticulomesenteric fibrous bands associated with intestinal mesentery or abdominal wall is enough in treatment of symptomatic Meckel's diverticulum; segmental ileal resection may be essential in treatment of complicated cases. There are different opinions on treatment of incidental (asymptomatic) Meckel's diverticulum [7].

In conclusion, awareness of the possibility of that there could be a gangrenous Meckel's diverticulum in a sac of strangulated umblical hernia has the mentionable importance and so, it may creates an innovation in the treatment and the postoperative rehabilitation modalities.

## Consent

The informed written consent was obtained from the patient for publication of this case report and accompanying images. A copy of the written consent is available for review by the Editor-in-Chief of this journal.

## Competing interests

The authors declare that they have no competing interests.

## Authors' contributions

All authors read and approved the final manuscript.
